# Script Training Using Telepractice with Two Adults with Chronic Non-Fluent Aphasia

**DOI:** 10.5195/ijt.2018.6259

**Published:** 2018-12-11

**Authors:** NAOMI C. RHODES, EMI ISAKI

**Affiliations:** DEPARTMENT OF COMMUNICATION SCIENCES & DISORDERS, NORTHERN ARIZONA UNIVERSITY, FLAGSTAFF, AZ, USA

**Keywords:** Chronic aphasia, Script training, Speech-language pathology, Telepractice

## Abstract

Two male participants with chronic (> 2 years), non-fluent aphasia and their family members participated in script training using videoconferencing. Functional scripts were developed by people with aphasia (PWA) and their family members. Accuracy of scripts was measured by total target words produced per turn. Participant 1 with Broca’s aphasia produced scripts with 0% accuracy pre-treatment and 87.5% accuracy post-treatment. Participant 2 with Transcortical Motor aphasia produced scripts with 20.2% accuracy pre-treatment and 63.5% accuracy post-treatment. Pre- and post-questionnaires for communication effectiveness and the use of telepractice for speech therapy indicated improvements in answering yes/no questions, participating in conversations with strangers, and increasing confidence and satisfaction with technology delivered treatment. The use of videoconferencing to deliver script training appears beneficial for individuals with chronic aphasia.

Each year approximately 795,000 individuals in the United States have a stroke or cerebrovascular accident (CVA), (Centers for Disease Control and Prevention [CDC], 2017). Of those diagnosed with a stroke or CVA, approximately 180,000 individuals will have aphasia (National Institute on Deafness and Other Communication Disorders [NIDCD], 2017). The NIDCD (2017) estimates that approximately one million Americans currently live with aphasia that can negatively affect verbal and non-verbal communication abilities. Speech-language pathologists assist in the rehabilitation of adults with aphasia in the areas of: anomia/word finding, expressive and receptive language, reading comprehension, writing, calculation, speech, and swallowing (American Speech-Language-Hearing Association [ASHA], 2017; Brookshire, 2015).

Of all the problems associated with aphasia, expressive language difficulties are most concerning to people with aphasia (PWA) and their communication partners. Mazaux et al. (2013) asked PWA one-year post-stroke to rate which everyday communication situations they found to be most difficult. PWA felt they struggled the most when using the phone for a meeting, using checks or credit cards, writing, communicating in conversations about complex themes, and interacting in social activities. In these difficult situations, script training was one method of treatment that provided PWA with the ability to participate in everyday, social communication situations involving automatic speech (Cherney, Kaye, Lee, & van Vuuren, 2015).

## SCRIPT TRAINING AND APHASIA

Scripts are mental schemata of routine communication situations in everyday life (Brookshire, 2015). Whether one works, interacts with friends and family, or participates in daily activities, certain preconceived expectations are placed on the speaker for what will occur and how to respond. These expectations and predictions aid in auditory comprehension and organization of information for communication success. Introductions to new people, listening to friends talk about their vacation, or asking someone where an item is located in the grocery store are examples of when scripts are used. Because aphasia can affect expressive and receptive language, any interaction from special occasions to routine activities can be difficult for PWA. Thus, the use of scripts may assist with communication difficulties for PWA in various situations.

Lee, Kaye, and Cherney (2009) analyzed the relationship between the amount of script training provided and participants’ improvement. Seventeen participants with non-fluent aphasia (*M* = 65.8 months post-injury) were recruited for the study. Therapy was conducted for approximately 12 weeks. The clinicians collaborated with PWA for two to three weeks to develop three scripts. Script topics included monologues and dialogues to convey information in restaurants, physician offices, and with family. Script complexity and number of turns varied according to aphasia severity. Scripts for less severe participants included up to 141 target words and 10 conversational turns with increased length. PWA received a laptop computer containing their recorded scripts and used AphasiaScripts software (Cherney, Halper, Holland, & Cole, 2008) to practice at home for nine weeks. Once each week, participants met in-person with a clinician for assistance and assessment of progress. For the 16 participants, production of words in their scripts improved by 45.72% and rate (i.e., the number of script-related words per minute) improved by 137.48%. When participants were divided into two groups by severity, those with less severe aphasia had less improvement in rate. Those with more severe aphasia spent extra time practicing, as measured by hours logged on to AphasiaScripts. In general, more treatment time led to greater gains. The authors concluded that future research should address differences in treatment intensity. Additionally, Cherney’s (2012) post hoc analysis suggested the best outcomes occur when treatment time focuses on sentence and conversation practice rather than words and phrases.

In another study, Holland, Halper, and Cherney (2010) paired 29 non-fluent PWA with clinicians to collaborate on script training. In some cases, a family member was involved. The participants had a mean age of 57.15 years, and mean *Western Aphasia Battery-Revised (WAB-R*; Kertesz, 2007) *Aphasia Quotient* (*AQ*) score of 53.72. All participants were at least six months post-injury. Three scripts were developed for each participant: a monologue, a dialogue with the PWA as initiators, and a dialogue with the PWA as respondents. Monologues were 10–15 sentences in length, and dialogues included eight to 10 turns. Thematic analysis of the participants’ 100 total scripts found the most prevalent monologue topics included retelling stories from their lives (i.e., pre-stroke life and the stroke event), stating their religious beliefs, and providing speeches. The 32 dialogues with PWA as respondents focused on interacting with salespeople, ordering in a restaurant, participating in hobbies, and making phone calls. The 40 dialogues with PWA as initiators included conversations with family, seeking or providing information to a variety of audiences, and expressing outside interests. Practice times varied from < 30 minutes per day up to 20 hours per week. Motivation and meaningfulness of script topics appeared to influence the participants’ amount of practice. Therefore, clinicians must consider themes that are meaningful and relevant to their clients. Although some PWA had family members involved in the session, perhaps a more functional approach would ensure that all PWA have a friend or family member involved in therapy.

Cherney et al. (2015) measured acquisition and generalization of personally relevant versus generic words and phrases. Eight participants with chronic aphasia (*M* = 26.4 months post-injury) were trained as respondents using script templates of a dialogue with 10 turns. All but one participant had non-fluent aphasia. Eight templates were developed (four shared items were personally relevant, and the others were generic) by the person with aphasia and the clinician. Spouses of PWA were consulted in cases of severe aphasia. AphasiaRx (van Vuuren & Cherney, 2014), an updated AphasiaScripts software, was loaned to PWA on laptops for practice at home for 90 minutes per day, six days a week for three weeks. A speech-language pathologist (SLP) visited weekly to ensure progress and observe practice; however, entire sessions were not observed. Accuracy for personally relevant and generic items were comparable at baseline. For trained scripts, PWA improved in both personally relevant and generic words. However, in untrained scripts testing for generalization, significant gains were seen only for personally relevant words and phrases. Findings from this study supported the importance of choosing personally relevant material for script training.

Developing individualized scripts with personally relevant material can be time consuming. To address this issue, Kaye and Cherney (2016) focused on the effects of varying the level of reading complexity for pre-determined script templates while inserting some personally relevant details (e.g., name of the town or a close acquaintance). Script templates in this study addressed PWA ordering in a restaurant and grocery shopping. Each script template was a dialogue of 10 turns across speakers. Readability was varied across syllables, words, sentences, grammatical morphemes, and frequency of use/semantic difficulty. Eight participants with chronic (*M* = 54.7 months post-injury) non-fluent aphasia (*WAB-R M =* 60.4) were assigned script levels for which they were 30% accurate at baseline. PWA were divided into two severity groups. The participants’ performance was assessed on scripts that were high and low levels of difficulty, which was defined as one level above or below relative to baseline level. Probe data were collected according to predetermined dates using AphasiaScripts software. Regardless of severity, PWA showed significantly greater accuracy for scripts of low difficulty, which indicates that clinicians should quantify difficulty of treatment to best support what is measured at baseline. PWA commented that they appreciated the personally relevant details of the scripts.

Given the communication difficulties associated with aphasia, script training provides a functional, social strategy for PWA to use in everyday interactions. In general, the best outcomes for script training occur with a greater amount of practice/speaking time (Cherney, 2012; Lee, Kaye, & Cherney, 2009), linguistic demands slightly above baseline, and personally meaningful topics (Cherney et al., 2015; Holland et al., 2010; Kaye & Cherney, 2016). In the literature reviewed, scripts were most often developed by PWA and a clinician, rather than PWA and their family members. Outcome measures used in prior studies have included production of script content words or phrases in various communication settings, and/or production of content words/or phrases per unit of time (Cherney et al., 2015; Lee et al., 2009). Content words in the previous studies referred to the total number of target words or independently meaningful words, such as nouns, verbs, adjectives, etc. To date, the script training studies completed mainly by Cherney focused on the use of the AphasiaScripts and AphasiaRx software programs. These virtual therapy programs were used primarily for homework presentation (van Vuuren & Cherney, 2014). Because AphasiaScript must be purchased, and AphasiaRx continues to be studied in research, the programs may not be readily accessible to PWA. Additional studies investigating different methods of service delivery for script training could prove beneficial. Finally, a collaboration between PWA, friend or family member, and clinician could assist in developing more functional scripts for more meaningful and individualized therapy.

### TELEPRACTICE

Telepractice, the remote provision of services via technology, was found to be a practical and convenient service delivery model because it overcomes geographic, transportation, and time commitment barriers, allowing for equal access to health care services (Bridgman, Onslow, O’Brian, Jones, & Block, 2016; Hall, Boisvert, & Steele, 2013; Woolf et al., 2016). Telepractice not only overcomes barriers to services, but also enhances generalization of treatment since the therapy is provided in the home environment (Bridgman et al., 2016; Goldberg, Haley, & Jacks, 2011).

Hall et al. (2013) conducted a systematic review of the literature regarding telepractice and aphasia assessment and/or treatment. Single-subject and multiple baseline studies were commonly conducted. Evaluation and therapy provided via telepractice found no significant differences between in-person and telepractice service delivery. In comparison to traditional, in-person therapy, telepractice improved interest in the stimuli, adherence to protocol, and attendance. Telepractice also resulted in more efficient use of time and overcame barriers of transportation, cost of therapy, and geographic barriers (Hall et al., 2013). Limitations of telepractice included internet and device connectivity, which resulted in signal delays and reduced quality of visual presentations. Additionally, some PWA were concerned about privacy and the complexity of the equipment used. Suggestions made by the authors for future studies included the use of more advanced technology for telepractice, and a wider range of assessments that include functional measures of treatment to further explore the advantages and disadvantages of telepractice.

Woolf et al. (2016) conducted a quasi-randomized feasibility study with PWA to compare remote therapy using FaceTime to in-person therapy in terms of treatment effectiveness, treatment fidelity, and compliance and satisfaction with technology. Twenty-one PWA, six months post-stroke, participated in research investigating anomia/word finding. PWA were required to choose their communication partner (i.e., friend, family member, or volunteer), and were trained to use an iPad and FaceTime technology. For four weeks, PWA were assigned to either FaceTime intervention provided from a university lab, FaceTime intervention from a clinical site, in-person therapy, or a control group of conversations held remotely. Intervention groups received picture naming therapy for one hour twice a week. Nineteen participants complied well and did not miss any of their sessions. All participants except one felt “good” about the technology. When difficulties with technology arose, PWA solved these by redialing or moving to another room. Six weeks after intervention, all participants were re-assessed. The greatest gains in conversational turns with a content word, content words per turn, and mean number of nouns per turn occurred for FaceTime therapy from a clinical site. Interestingly, the least amount of change during the study occurred for the FaceTime therapy provided from the university lab.

The goal of any aphasia therapy is generalization, which is defined as evidence that skills gained in therapy have carried over into untrained tasks (Brookshire, 2015; Cherney et al., 2015). Goldberg, Haley, and Jacks (2012) investigated the use of script training to improve measures of speech production (i.e., accuracy, grammatical competence, rate of speech, articulatory fluency), examine effects of script training with a new partner in conversations that did and did not adhere to scripts, and assess the service delivery model of videoconferencing combined with in-person sessions. Two case studies involving PWA, at least five years post-injury, with differing fluency abilities were described. One participant had a traumatic brain injury and Broca’s aphasia (*WAB-R AQ* = 57.2), and the other had conduction aphasia (*WAB-R* = 70.5). In this multiple baseline treatment design, a pre-baseline probe was taken in conversation to determine approximate script length. Next, a minimum of three baseline probes were taken, about two to three days apart, until performance was stable for at least one of the measures. The participants collaborated with their family members across two sessions to create two dialogue scripts that addressed everyday situations in which they desired better communication, such as general interests, experiences, and values. Ideas for script topics were provided based on themes found to be important to PWA by Holland et al. (2010). The participants were instructed not to practice during the baseline probing phase. For three weeks, PWA received script training three times per week either in-person or using videoconferencing. In-person therapy consisted of training one line of the script at a time while progressing through a cueing hierarchy that ended with independent production. PWA agreed to practice independently for 15–30 minutes, five days a week and were provided with a recording of the clinician producing the script. Generalization probes were taken after the second script had been maintained for one week. Participants then communicated with a new conversation partner who knew the topic of the treatment script. The participants engaged in a conversation with either the new partner or the clinician using pertinent, novel scripts. Both participants improved in script performance. The less severe participant improved on all measures, while the participant with traumatic brain injury and Broca’s aphasia improved in accuracy, grammatical competence, and articulatory fluency while rate of speech continued to improve beyond the maintenance phase. Disfluencies per word, which were mostly self-revisions, did not decrease. The participants used their scripts to support spontaneous utterances and introduce new conversation topics. The authors found videoconferencing combined with in-person therapy supported generalization of script training.

Although positive changes were reported, Beck (2012) noted several weaknesses in the Goldberg et al. (2011) study. Beck indicated that Goldberg et al. did not specify how they ensured proper implementation of the study under time limitations and difficulties with videoconferencing. Time limitations resulted in the collection of fewer than five data points per phase and prevented one from knowing whether conclusions would have been the same if participants were expected to meet an established criterion. In addition, Goldberg’s measurements of generalization were problematic because the baseline probes were unique from the generalization probes. Beck found problems with the generalization probes because the novel conversation partners were familiar with the topic of the script.

Finally, Snook (2013) used the telephone to measure the effectiveness and generalization of script training, and to obtain quality of life measures for two PWA and their family members in a multiple-baseline design. Both PWA were six months post-stroke, received scores higher than 40 on the *WAB-R Repetition* subtest, and were moderate in severity for dysarthria and/or apraxia of speech. One participant had mild anomic aphasia (*WAB AQ* = 81.6), and the other had moderate to severe Broca’s aphasia (*WAB AQ* = 48.2). For two to three weeks before intervention, PWA, family members, and the clinician collaborated to develop three scripts. Once scripts were finalized, baseline data were collected over the phone by graduate clinicians prompting PWA to produce their scripts. Script training occurred over the phone three to four times per week for an average of 10–25 minutes per session. Script mastery was defined as independent production with 90% accuracy as calculated by script words divided by total words produced. Four weeks following intervention, maintenance probes were collected over the phone. Generalization probes were collected at the beginning of every therapy session and weekly during in-person group therapy in response to the clinician’s conversational prompt. One participant and spouse lacked data for maintenance follow-up. PWA improved in their performance of scripts, but the skills did not generalize to conversation or novel conversations in familiar settings. PWA felt somewhat positive about script training on quality of life measures, but scores were comparable to their pre-treatment ratings. Limitations of this study were lack of visual cues available through videoconferencing and not investigating script training effects on functional communication outcomes.

Overall, telepractice services appear to be equivalent in effectiveness to in-person therapy. Telepractice has the potential for greater ecological validity since therapy is provided in more natural communication environments than a clinic. Advances in quality and ease of use are positive features of current technology. Regarding script training and telepractice, more studies are needed that investigate therapy with PWA for functional, conversation-level tasks. Therefore, the overall aims of the current pilot study were:

1. To determine if functional communication outcome scores change on the Communicative Effectiveness Index pre- and post-script training therapy for PWA and their family members.
2. To determine if opinions about telepractice change following script training as measured on a pre- and post-researcher developed telepractice questionnaire for PWA and their family members.
3. To determine if PWA improve in accuracy of script training using videoconferencing as measured by mean content words (i.e., target words correct) per turn.

## METHODS

### PARTICIPANTS

Two adult males (37 and 66 years) with severe apraxia of speech (AOS) and chronic, non-fluent aphasia (*M* = 4 years post-injury, range 3–5 years) were recruited from the Speech-Language-Hearing Clinic at Northern Arizona University (NAU) to participate in this research study. Prior to the study, the participants attended aphasia group therapy for approximately 2 years. Participant 1 had Broca’s aphasia, and Participant 2 had Transcortical Motor (TCM) aphasia. Additionally, a family member (i.e., a parent for Participant 1 and a spouse for Participant 2) participated in the study to assist with the videoconferencing program, and complete pre- and post-therapy questionnaires.

### MATERIALS

Two institutional review board (IRB) approved consent forms, one for PWA and the other for their family members, were reviewed and signed by the participants. Next, the PWA and their family members completed questionnaires regarding overall communication with the *Communicative Effectiveness Index* (*CETI*) (see Appendices A & B). The *CETI* is a 16-item questionnaire that asks about functional communication abilities, not just linguistic capabilities (Lomas et al., 1989). The CETI was modified for PWA by using a 3-point rating scale with = 1 (good), 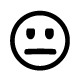 = 2, or = 3 (bad) for responses. The family member used the original version of the CETI with 1= strongly agree to 5 = strongly disagree. Larger scores indicated more negative feelings associated with each statement.

Similar rating scales were completed for a researcher-developed telepractice questionnaire. The telepractice questionnaire was developed from qualitative findings in the literature about familiarity and satisfaction using videoconferencing. Satisfaction with telepractice was measured for PWA and their family members (see Appendix C & D). Satisfaction was based on participant attitudes, perceptions, knowledge, and ease of use with videoconferencing.

In order to determine severity and type of aphasia, the *Western Aphasia Battery – Revised Aphasia Quotient* (*WAB-R AQ*) subtests were administered to PWA. This assessment evaluates linguistic and non-linguistic skills commonly affected by left hemisphere strokes.

Scripts were developed by PWA and the family members for important, everyday communication interactions. The primary researcher assisted in simplifying the selection of words in the script as needed. However, PWA and family members were always given choices for the words that were simplified in the script, and they determined the final words used.

The videoconferencing software was used on a desktop computer for Participant 1 and on an iPad for Participant 2. The primary researcher used a laptop computer to provide the script training. All targeted scripts were typed and sent to the participants via email. Finally, paper and pencil were used to take notes during script training sessions.

**Table:** Scripts for Participant 1

Hi, Doc. How are you?
Thank you, *.
I had a stroke.
My name is *.
I need help.
No, thank you.

Note:

* Removed for confidentiality purposes

**Table:** Scripts for Participant 2

My name is *. What is your name?
When do we leave?
I love you. Happy Valentine’s Day.
Please get my cup.
Go to bathroom

Note:

* Removed for confidentiality purposes

### PROCEDURES

Prior to therapy, the primary researcher administered the *WAB-R AQ* subtests to each person with aphasia to determine the aphasia type and severity. Next, the PWA and their family members completed the *CETI* and telepractice questionnaires. When needed, questionnaire items were read aloud, repeated, and broken into shorter segments to ensure that PWA comprehended the material. Finally, the primary researcher ensured that all participants could access the researcher and were comfortable using the videoconferencing program in order to complete treatment. The PWA and their family members developed three scripts for functional situations in which communication was most frustrating.

In the first session, telepractice was implemented by the primary researcher to finalize script development and collect baseline data. Thirteen sessions were completed with therapy occurring two times a week for 45 minutes. All sessions were audio-recorded for data analysis. At the beginning of each session, baseline data were collected, and the primary researcher asked participants whether they had practiced the scripts as homework. Three scripts were targeted throughout the study, rather than being taught sequentially. In script training, the primary researcher read the script aloud for the PWA to repeat. The PWA listened and watched the primary researcher. Participant 1 preferred phonemic cues and word segmentation, while Participant 2 requested whole word repetition and increased intonation. Cues were decreased as the PWA improved in their productions. Finally, the primary researcher provided a variety of questions pertaining to the scripts that required each person with aphasia to respond with the target scripts. For example, “If you visit your doctor, what could you say?”

After every therapy session, homework was assigned to the PWA and their family members. The family members cued the PWA to produce a script and let the individual practice the scripts as much as possible. Homework was expected to be completed daily at a convenient time. Within one week of each session, the data were transcribed, and mean content words per turn were calculated. All audio-recordings of the sessions were deleted after one week. New phrases were introduced upon mastery (i.e., verbatim production without cues for two consecutive sessions) of targeted phrases.

During the two weeks (sessions 13–16) of no script training, PWA engaged in Promoting Aphasics Communication Effectiveness (PACE) therapy (Davis & Wilcox, 1985) without any script training practice. Both participants were familiar with this therapy from previous aphasia group sessions that focused on maintaining communication skills. PACE requires both the participant and researcher/clinician to be the sender and receiver of information during communication. Colored photographs of actions and objects were selected as stimulus items. Questions were asked about the stimulus, and responses to the questions were provided to determine what was pictured. All modes of communication were used during the interaction. During the two weeks of PACE therapy, the PWA could practice scripts at home with no feedback from the primary researcher. Baseline data were collected at the beginning of session 13, before PACE was implemented, and then final data were collected at the end of session 16. In session 16, the PWA were asked to produce their scripts in response to the primary researcher’s questions over videoconferencing, and then all participants completed the post-therapy *CETI* and telepractice questionnaire in-person.

### DESIGN OF STUDY AND DATA ANALYSIS

This eight-week study included a multiple-baseline, ABCA, single-subject design. Pre- and post-mean score differences were compared on the *CETI* and telepractice questionnaire. For the script training, the start of therapy was staggered across participants. During the treatment phase, scripts were trained simultaneously, rather than sequentially. Therapy outcomes for script training were determined by accuracy as measured by mean content words per turn. Because non-fluent aphasia and apraxia of speech may have prevented the production of sentences, the number of content words (i.e., target words correct) out of all words in the scripts, were coded. To account specifically for apraxia of speech, approximated productions that could be understood by an unfamiliar listener were coded as correct and unintelligible utterances were coded as incorrect. Data for accuracy were plotted to track and analyze changes. During the no treatment sessions, PACE was used to maintain communication skills, without any focus on scripts.

## RESULTS

### SPECIFIC AIM 1

To determine if functional communication outcome scores change on the *Communicative Effectiveness Index (CETI)* pre- and post-script training therapy for PWA and their family members.

Overall the participants’ scores decreased on the *CETI* indicating fewer negative perceptions about communication abilities post-treatment (see Tables 1 & 2). Average *CETI* scores reported by PWA decreased from 28.5 to 24.5 (see Table 1). PWA did not have a common decrease in any one item. Family members reported an average change from 48 to 37 for PWA on the *CETI*. Questions 11 & 15 on the family-reported *CETI* had the most changes, and addressed the ability to communicate anything (including yes/no) without words and participating in conversation with strangers.

Participant 1’s *CETI* scores decreased from 29 to 22 (Table 1). The item that changed the most (from 3 to 1) was the ability to start a conversation with people who are not close family. He improved by one point for Questions 4, 5, 7, 10, 15, and 16. Participant 1’s family member also completed the *CETI* with a decrease in score from 52 to 41 (Table 2). The family member indicated the most improvement was in Participant 1’s ability to respond to or communicate anything (including yes/no) without words (decrease from 4 to 1). According to the family member, he also improved in his abilities to indicate that he understood what is being said to him, engaging in conversations with friends and neighbors, and participating in a conversation with strangers.

*CETI* scores of Participant 2 decreased from 28 to 27 (Table 1). Participant 2 reported that he felt better about communicating physical problems such as aches and pains (decrease from 3 to 2) and giving yes/no answers appropriately (decrease from 2 to 1). The *CETI* score of Participant 2’s family member decreased from 44 to 33 (Table 2), with the most changes in ability to communicate physical problems such as aches and pains (decrease from 4 to 2) and being part of a conversation when it is fast and there are a number of people involved (decrease from 5 to 3).

However, scores for some items on the *CETI* increased pre-and post-treatment indicating more negative feelings. Participant 1 felt that he decreased in his ability to get involved in group conversations about himself (increase from 1 to 2). Participant 1’s family member felt this had improved (decrease from 4 to 2), but reported more negative feelings about his ability to say the name of someone whose face was in front of him (increase from 4 to 5) and starting a conversation with people who were not close family (increase from 3 to 4). Participant 2 felt he had worsened for indicating that he understood what was being said to him (increase from 1 to 2), while his family member felt that he had remained the same for this item but was worse at getting somebody’s attention (increase from 1 to 2).

### SPECIFIC AIM 2

To determine if opinions about telepractice change following script training as measured on a pre- and post-researcher developed telepractice questionnaire for PWA and their family members.

Average telepractice questionnaire scores decreased from 14.5 to 8.5 for the PWA and from 21 to 8.5 for their family members (see Tables 3 & 4). The greatest changes in perception occurred for familiarity/ease of use for telepractice technology and recommending telepractice therapy to a friend. All participants felt more familiar with telepractice post-therapy, as indicated by a decrease in average scores from 2.5 to 1.5 for PWA and 5 to 1.5 for family members. They were more likely to agree with the statement that telepractice was equivalent to in-person therapy. Similarly, all participants reported they would recommend telepractice therapy to a friend with post-treatment scores decreasing to 1 = yes/strongly agree.

Participant 1’s telepractice scores decreased from 14 to 8. He felt positively (scores of 1) about being familiar with telepractice, thought that therapy using telepractice was the same quality as in-person therapy, was comfortable using his computer for videoconferencing, and felt that telepractice was convenient for him. His rating of satisfaction with the visual signal did not change pre-and post-therapy (score of 2). His family member reported similar ratings. The family member’s perception of telepractice was equivalent to in-person therapy as a rating of 3 (neutral) changed to 1 (agree).

Participant 2’s telepractice scores decreased from 15 to 9. Post-treatment scores of 1 were reported for ease using his iPad for videoconferencing, the visual signal, and telepractice convenience. He gained familiarity with telepractice (score changing from 3 to 2) and felt neutral about the quality of telepractice as compared to in-person therapy (pre- and post-treatment score of 2). Participants 2’s family member rated all items as 1 on the post-treatment telepractice questionnaire.

### SPECIFIC AIM 3

To determine if PWA improve in accuracy of script training using videoconferencing as measured by mean content words (i.e., target words correct) per turn.

At the beginning of every session, the primary researcher asked each person with aphasia if he had practiced his scripts or completed assigned homework. At least > 50% of the time, PWA indicated that they had not. Participant 1 practiced more than Participant 2. Both PWA improved in their script production accuracy. Participant 1 with Broca’s aphasia improved from 0% accuracy to an average of 87.5% accuracy upon final data collection (Table 5). Participant 2 with TCM aphasia had an increase in average accuracy from 20.2% to 63.5%. Participant 1 demonstrated mastery of his scripts (i.e., produced scripts verbatim with no cues over two consecutive sessions). He also learned new scripts throughout the study. Overall, Participant 1 mastered four scripts and produced two scripts with phonemic cues for a total of six scripts used in treatment.

**Figure 1:**
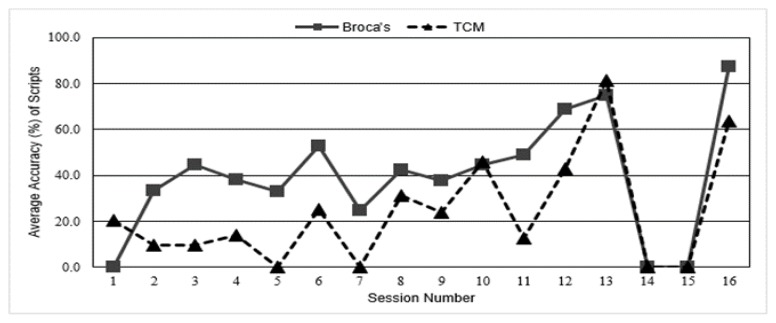
Average percent accuracy of baseline data collected during 13 sessions of script training therapy. Average accuracy (%) of scripts per session as measured in words correct out of words total. Baseline data for Participant 1 with Broca’s aphasia and Participant 2 with Transcortical Motor aphasia are presented for each session. No script training was delivered for two weeks, sessions 13–16. The baseline data for session 13 were collected at the beginning of session. Final data were collected at the end of session 16.

Participant 2 worked on the same four scripts for the duration of the study. Participant 2 demonstrated mastery within session but was unable to produce accurate scripts independently at the start of therapy sessions when data were collected (see Figure 1).

Finally, anecdotal findings suggest that both participants continued to generalize the scripts trained in the research project. Following completion of the research study, the participants attended aphasia therapy for four weeks. When asked questions related to the scripts, PWA often produced the scripts spontaneously or required phonemic cues to begin the script. Both PWA seemed very excited that they could respond to the questions asked of them.

## DISCUSSION

In speech-language pathology, script training has been used with PWA who have difficulty with expressive language and/or speech production. The use of script training can enhance interactions by improving the production of common words, phrases, or sentences in daily functional activities such as greeting friends or family members, meeting new people, or asking for help. Generalization for script training occurs when PWA can use the scripts during everyday conversations both inside and outside the home environment.

Prior studies investigating script training have found positive results in the production of scripts with PWA who have used the AphasiaScript and newer AphasiaRx software programs (Cherney et al., 2015; Kaye & Cherney, 2016; Lee, Kaye, & Cherney, 2009; van Vuuren & Cherney, 2014). Additionally, prior studies reported that the scripts were often developed by the clinician and the person with aphasia, rather than the person with aphasia and a family member (Cherney et al., 2015; Kaye & Cherney, 2016; Lee, Kaye, & Cherney, 2009). Thus, the question remains whether the scripts were truly functional to the PWA in their daily activities.

The current study investigated script training with two PWA by providing telepractice services using videoconferencing software on their preferred technological devices. Videoconferencing allowed the primary researcher and PWA to participate in synchronous (real-time) script training with models of productions provided by an actual clinician. The PWA and their family members selected topics and developed scripts for daily functional activities. The primary researcher provided suggestions to simplify some of the wording used in the scripts if the words were polysyllabic or included sound blends that were difficult to produce by PWA. Additionally, pre- and post-therapy questionnaires about communication outcomes and perceptions about telepractice were completed by the participants. The measures were used to provide a better understanding of how script training provided by technology affected these areas

Script training provided with videoconferencing indicated some gains in communication as measured by the *Communication Effectiveness Index* (*CETI*). All participants felt fewer negative feelings about PWA’s communication ability overall; however, improvement in specific communication abilities were not always the same across participants. The participants felt most positively about PWA communicating anything (including yes/no) without words and participating in conversations with strangers. Increased participation with strangers is a positive outcome supported by the script training literature as the intent is to promote increased functional and social communication.

Regarding telepractice, participants rated therapy via videoconferencing positively and attended every session. These results are similar to the findings of Woolf et al. (2016). Prior to beginning the therapy, participants were hesitant about videoconferencing because of technology concerns and the possible reduction of cues provided with telepractice. Following treatment, the participants thought that the use of videoconferencing was convenient for them since they stayed in their homes, used familiar devices, and did not have to travel. These reasons are consistent with the findings of Bridgman et al. (2016) and Hall et al. (2013) who reported similar patient-perceived benefits of telepractice. Family members reported that they felt therapy was effective because PWA received therapy twice a week, rather than once a week. Interestingly, while not all participants felt that telepractice was equivalent to in-person therapy, they stated that they would recommend it to a friend.

Both PWA improved in script production (which appears to be associated with frequent practice), conversation-based therapy, targets with linguistic demands slightly above baseline, and scripts that were personal and functional to their lives. These findings are consistent with the work and recommendations of Cherney (2012), Cherney et al. (2015), Holland et al. (2010), Kaye and Cherney (2016), and Lee et al. (2009). Participants were highly motivated to produce their scripts to introduce themselves to others, greet healthcare professionals, indicate their needs, and thank family members or caregivers.

Although both PWA increased in their abilities to produce scripts, Participant 1 with Broca’s aphasia produced a greater number of scripts that were mastered and often generalized within and across sessions. Participant 2 with Transcortical Motor aphasia did not generalize his scripts across sessions and often required a model of the entire script or the beginning word of the script. Transcortical Motor aphasia is similar to Broca’s aphasia but is characterized by good repetition abilities. Theoretically, repetition skills should have been advantageous in script training for Participant 2. Initially, the participants relied heavily on repetition of the script that was modeled by the primary researcher. The problems occurred when the verbal and visual cues were decreased. Thus, generalization of scripts did not occur as frequently for Participant 2 as it did for Participant 1. Participant 2 required several productions of the script by the primary researcher before he could produce it with fewer cues. During script training, both participants exhibited perseveration, which occurred as words produced from the previous script. Participant 1 often had fewer perseverations than Participant 2.

Another finding was that the participants had different practice styles and environments. Participant 1 with Broca’s aphasia had family present, but not directly involved in therapy. Participant 2 with Transcortical Motor aphasia often relied on his family member to adjust the telepractice technology. The family member also provided in-person cues after the clinician’s model.

Participants also preferred and benefitted from different types of cueing. Participant 1 with Broca’s aphasia preferred a slower rate of production of words so that he could watch the clinician’s speech motor movements. Participant 2 with Transcortical Motor aphasia benefitted from regular speech rate with increased prosody. Thus, therapy that was tailored to the person with aphasia’s needs assisted in script training. Consistent with the findings of Kaye and Cherney (2016), the scripts targeted in therapy of lower difficulty were easier to master. For instance, both participants struggled with polysyllabic words and words with consonant blends. Once these scripts were revised to be simpler, success increased. PWA and their family members were always given choices of the simplified words used in the scripts.

Script themes chosen by participants were consistent with the results of Holland et al. (2010). Participants’ scripts prioritized being able to introduce themselves, interact with service providers, and converse with family. In contrast, participants did not choose scripts that discussed religion, hobbies, or telephone use. Script topics were less abstract and focused on activities of daily living such as communicating the need for help and requesting items.

Although interesting findings were noted in the study, several limitations were evident. First, the no treatment period in this study was shorter than prior studies (Cherney, Kaye, Lee, & van Vuuren, 2015; Snook, 2013; van Vuuren & Cherney, 2014). Typically, these studies incorporated between three to four weeks of no treatment before final data were obtained. Another limitation was the fidelity of the videoconferencing signal. Although the audio signal continued to work without difficulty, the video image froze approximately one to three times per session when using videoconferencing. However, PWA became very adept at ending the call and contacting the primary researcher for a new videoconferencing call. This same solution was reported by participants in the Woolf et al. (2016) study.

## CONCLUSION

This study investigated the perceptions of two PWA and their family members regarding communication and telepractice for script training. Functional outcome measures appeared to be commensurate with improved accuracy for script production. All participants improved not only in script production, but also in attitudes toward telepractice. Those who improved the most perceived the most benefit from the therapy. Future research should conduct group studies using telepractice and include people with various types and severities of aphasia, continue to compare the differences and similarities of telepractice versus in-person therapy, and investigate communication strategies that optimize telepractice services. As advances in technology occur, clinicians will have more choices in the software programs used. Therefore, the use of telepractice to provide services for aphasia is expected to improve and expand in the field of speech-language pathology.

## Figures and Tables

**Table 1:** Communication Effectiveness Index Scores for People with Aphasia

	Participant 1		Participant 2	
Question	Pre	Post	Pre	Post
Getting somebody’s attention.	1	1	1	1
Getting involved in group conversations that are about him/her.	1	2	2	2
Giving yes and no answers appropriately.	1	1	2	1
Communicating his/her emotions.	2	1	1	1
Indicating that he/she understand what is being said to him/her.	2	1	1	2
Having coffee-time visits and conversations with friends and neighbors (around the bedside or at home).	1	1	1	1
Having a one-to-one conversation with you.	2	1	1	1
Saying the name of someone whose face is in front of him/her.	3	3	2	2
Communicating physical problems such as aches and pains.	1	1	3	2
Having a spontaneous conversation (i.e., starting the conversation and/or changing the subject).	3	2	2	2
Responding to or communicating anything (including yes or no) without words.	1	1	1	1
Starting a conversation with people who are not close family.	3	1	2	2
Understanding writing.	2	2	2	2
Being part of a conversation when it is fast and there are a number of people involved.	1	1	3	3
Participating in a conversation with strangers.	2	1	2	2
Describing or discussing something in depth.	3	2	2	2
Total	29	22	28	27

**Table 2:** Communication Effectiveness Index Scores for People with Aphasia by Family Member

	Participant 1		Participant 2	
Question	Pre	Post	Pre	Post
Getting somebody’s attention.	1	1	1	2
Getting involved in group conversations that are about him/her.	3	2	3	3
Giving yes and no answers appropriately.	2	2	3	2
Communicating his/her emotions.	2	1	3	2
Indicating that he/she understand what is being said to him/her.	4	2	2	2
Having coffee-time visits and conversations with friends and neighbors (around the bedside or at home).	4	2	2	2
Having a one-to-one conversation with you.	3	2	2	1
Saying the name of someone whose face is in front of him/her.	4	5	3	2
Communicating physical problems such as aches and pains.	3	3	4	2
Having a spontaneous conversation (i.e., starting the conversation and/or changing the subject).	2	2	2	2
Responding to or communicating anything (including yes or no) without words.	4	1	2	1
Starting a conversation with people who are not close family.	3	4	3	2
Understanding writing.	4	4	3	2
Being part of a conversation when it is fast and there are a number of people involved.	5	5	5	3
Participating in a conversation with strangers.	5	3	3	2
Describing or discussing something in depth.	3	2	3	3
Total	52	41	44	33

**Table 3:** Telepractice Questionnaire Scores for People with Aphasia

	Participant 1		Participant 2	
Question	Pre	Post	Pre	Post
I am familiar with telepractice.	2	1	3	2
I think therapy using telepractice is the same quality as in-person therapy.	2	1	2	2
I am comfortable using my computer/laptop/tablet.	1	1	1	1
The video conferencing software program is easy for me to use.	2	1	2	1
The visual signal was satisfactory.	2	2	2	1
Telepractice is convenient for me.	2	1	3	1
I would recommend telepractice therapy to a friend	3	1	2	1
Total	14	8	15	9

**Table 4:** Telepractice Questionnaire Scores for Family Member

	Participant 1		Participant 2	
Question	Pre	Post	Pre	Post
I am familiar with telepractice.	5	2	5	1
I think therapy using telepractice is the same quality as in-person therapy.	3	2	3	1
I am comfortable using my computer/laptop/tablet.	3	1	2	1
The video conferencing software is easy for me to use.	3	1	3	1
The visual signal was satisfactory.	2	2	3	1
Telepractice is convenient for me.	1	1	3	1
I would recommend telepractice therapy to a friend	3	1	3	1
Total	20	10	22	7

**Table 5:** Average Percent Accuracy of Baseline Data Collected During 13 Sessions of Script

Training Therapy		
Session	Participant 1	Participant 2
1	0.0	20.2
2	33.3	9.8
3	44.4	9.8
4	38.3	14.0
5	32.8	0.0
6	52.9	25.0
7	24.6	0.0
8	42.5	31.3
9	37.5	24.0
10	44.6	45.8
11	48.8	12.5
12	68.8	42.7
13	75.0	81.3
14	0.0	0.0
15	0.0	0.0
16	87.5	63.5
